# Metagenomic Profiling of a Microbial Assemblage Associated with the California Mussel: A Node in Networks of Carbon and Nitrogen Cycling

**DOI:** 10.1371/journal.pone.0010518

**Published:** 2010-05-06

**Authors:** Catherine A. Pfister, Folker Meyer, Dionysios A. Antonopoulos

**Affiliations:** 1 Department of Ecology and Evolution, University of Chicago, Chicago, Illinois, United States of America; 2 Computation Institute, University of Chicago, Chicago, Illinois, United States of America; 3 Institute for Genomics and Systems Biology, Argonne National Laboratory, Argonne, Illinois, United States of America; 4 Department of Medicine, University of Chicago, Chicago, Illinois, United States of America; American Museum of Natural History, United States of America

## Abstract

Mussels are conspicuous and often abundant members of rocky shores and may constitute an important site for the nitrogen cycle due to their feeding and excretion activities. We used shotgun metagenomics of the microbial community associated with the surface of mussels (*Mytilus californianus*) on Tatoosh Island in Washington state to test whether there is a nitrogen-based microbial assemblage associated with mussels. Analyses of both tidepool mussels and those on emergent benches revealed a diverse community of Bacteria and Archaea with approximately 31 million bp from 6 mussels in each habitat. Using MG-RAST, between 22.5–25.6% were identifiable using the SEED non-redundant database for proteins. Of those fragments that were identifiable through MG-RAST, the composition was dominated by Cyanobacteria and Alpha- and Gamma-proteobacteria. Microbial composition was highly similar between the tidepool and emergent bench mussels, suggesting similar functions across these different microhabitats. One percent of the proteins identified in each sample were related to nitrogen cycling. When normalized to protein discovery rate, the high diversity and abundance of enzymes related to the nitrogen cycle in mussel-associated microbes is as great or greater than that described for other marine metagenomes. In some instances, the nitrogen-utilizing profile of this assemblage was more concordant with soil metagenomes in the Midwestern U.S. than for open ocean system. Carbon fixation and Calvin cycle enzymes further represented 0.65 and 1.26% of all proteins and their abundance was comparable to a number of open ocean marine metagenomes. In sum, the diversity and abundance of nitrogen and carbon cycle related enzymes in the microbes occupying the shells of *Mytilus californianus* suggest these mussels provide a node for microbial populations and thus biogeochemical processes.

## Introduction

In many locales in coastal oceans, nitrogen has been demonstrated to be the limiting nutrient, with large-scale circulation patterns (such as upwelling) being the primary determinant of coastal productivity. Although circulation patterns that drive upwelling can import substantial amounts of nitrate into coastal areas, regeneration of nitrogen *in situ* can also contribute to local productivity [1] [Bibr pone.0010518-Eppley1]–[3]. Regenerated nitrogen is mostly due to the metabolism and excretion of animals, while marine plants, seaweeds and microbes utilize the nitrogenous waste. Although the response of some coastal eukaryotic primary producers to nitrogen production by animals has been described [4] [Bibr pone.0010518-Bracken1]–[6], microbial population abundance and diversity in response to nitrogen is less studied. Nonetheless, there is ample evidence that microbes are ubiquitous consumers of nitrogeneous byproducts from animals, chemolithotrophy is well-established, and there is a great potential for regenerated nitrogen availability to drive enhanced carbon dioxide fixation.

Despite the importance of nitrate delivery with upwelling along the margins of northeast Pacific Ocean, ammonium excretion by animals is detectable [7]–[10] and has been shown to contribute to local productivity [5], [6], [10] and diversity [11]. Although marine mammals, seabirds, fishes and dense aggregations of invertebrates all may contribute to regenerated nitrogen in coastal areas, mussels (*Mytilus californianus*, henceforth mussels) have only recently been recognized as significant contributors [6], [10]. Experimental manipulation of the presence of mussels demonstrated that ammonium excretion by invertebrates not only boosts the productivity of macroalgae, but also drives microbial productivity via nitrification [6]. The use of animal-regenerated nitrogen for chemolithotrophy by marine microbes has been relatively ignored in these well-studied rocky shores; arguably, their abundance and function is probably better understood in the open ocean [12],[13] and deep sea environs [14]. To date, we know relatively little about the identity or function of rocky shore microbes and their importance to nitrogen and carbon cycling. Marine benthic nearshore microbes may play an important role mediating the abundance of different forms of nitrogen via nitrification, ammonification, detnitrification and potentially all aspects of the nitrogen cycle. Additionally, they are likely providing increased opportunities for microbial CO_2_ fixation, while also competing with other primary producers, including the ecologically important macroalgae, for nitrogen. Here we describe shotgun metagenomic-based analysis of the microbes associated with mussels including analyses of their function in rocky shore ecosystems.

It is thought that many microbial taxa cannot be cultured outside of their natural environment; thus, microbial diversity remains poorly described [15], [16]. The metagenome techniques developed recently have therefore greatly extended our knowledge of microbial genetic diversity [17] [Bibr pone.0010518-Dinsdale1]–[19]. Because they are acclimated to high energy waves and cold temperatures, many rocky shore species, including microbes, are difficult to accommodate in laboratory environs. The recent findings of the previously undescribed nitrifying Archaea in a diversity of habitats [20] [21], suggest that there is much microbial diversity yet to be described. Additionally, the ability to analyze vast numbers of genomes allows probable metabolic functions to be determined [22]. Because we had strong experimental evidence that microbial nitrification was present in tidepools with abundant mussels [6], we hypothesized that these microbes would live in close proximity to a reliable source of both habitat and ammonium – the shells of the mussels themselves. We further hypothesized microbial assemblages would be common to mussels in a variety of habitats on rocky shores, due to their dominance and abundance [23], [24]. Indeed, mussels average densities of mussels are 4661 per m^2^ on Tatoosh Island [25], the site of the work reported here. We thus report metagenome analyses of the microbial community obtained from shells of mussels, including separate analyses of the community from tidepool mussels versus those from mussels that reside on rock that is emergent at low tide. Specifically, we ask about the taxonomic affiliations of these microbial communities as well as the likely function of these microbes given their affiliations and their sequence homology with enzymes of known function in nitrogen metabolism.

The increasing public availability of environmental metagenomes has further allowed us to compare our mussel microbial assemblage both in terms of taxonomy and metabolism to other ecosystems. We further use results from other marine ecosystems to test whether mussel-associated microbes have similar nitrogen-based metabolism.

## Materials and Methods

Mussels were collected from the Main Beach site of Tatoosh Island (48.32°N, 124.74°W), located in the eastern Pacific 0.7 km off the northwestern tip of Washington State, USA. Six mussel shells were collected from among 6 tidepools, while 6 more were collected at a distance of approximately 5 m apart on an adjacent exposed bench on 10 April 2008 and immediately cleaned of all soft tissue. The shells (mean length 4.47 cm and 4.42 cm for tidepool and bench mussels respectively) were put on ice and brought to Argonne National Labs.

DNA was extracted and purified using Ultraclean Mega Prep Soil DNA Isolation Kit and following directions therein (MO BIO Laboratories,Inc.) and the two extractions are referred to as tidepool versus bench mussels. The tidepool sample yielded 4320 ng in 108 µL (Invitrogen Qubit fluorometer dsDNA HS Kit), while bench mussels had 168 ng in 350 µL and required use of the GenomiPhi V2 DNA Amplification Kit (GE Healthcare). We followed the Roche GS-FLX (454) shotgun library preparation protocol; the tidepool sample used 2.4 µg and the bench sample used 5.0 µg for library preparation. Both samples had a mean fragment size of 750 bp after library preparation. All sequencing was performed with the 454 GS-FLX instrument and LR70 sequencing chemistry (Roche Applied Science).

We analyzed the taxonomic composition of our two metagenome sample sets with the MG-RAST server [26] using similarity to a large non-redundant protein database. Using the same non-redundant database, we also tested the affinities of our sequences for known metabolic function against both SEED subsystems [27] and KEGG metabolic pathways [28] using a maximum e-value of e<10^−5^. Although there are a number of metabolic functions that can be tested, our specific interest in microbial contributions to the nitrogen cycle focused our efforts on both nitrogen metabolism and carbon dioxide fixation. Thus, we probed particularly for enzymes related to the components of nitrogen and CO_2_ use.

In addition to describing the taxonomic and metabolic features of this microbial community on mussels, we also tested the similarity and differences with other recently described marine microbial assemblages that are public, including those of coastal Georgia [29], 4 tropical Pacific Ocean seawater samples in the Line Islands [19], and the extensive Global Ocean Sampling Expedition [13]. For the latter, we chose for comparison 4 coastal locales that that spanned a wide geography and sampled surface waters, including the Gulf of Maine (GS002, MG-RAST id #4441579.3), Nag's Head, NC (GS013, 4441585.3), Cocos Island, Costa Rica (GS025, 4441593.3), and an upwelling zone off of Fernandina, Galapagos (GS031, 4441597.3). We excluded marine metagenome analyses that had selectively filtered and extracted samples to isolate viruses. We focused our analyses on nitrogen metabolism and CO_2_ fixation to test the similarities and differences of our mussel-associated microbes. Given the abundance of nitrogen in our mussel-associated waters, we further asked if another nitrogen-rich ecosystem, soils of the agriculture-influenced midwest, showed metabolic similarities. Here, we compared our mussel microbial assemblage to soil samples from Midwestern locales (Waseca farm soil (4441091.3), soybean field (4442657.3), prairie remnant (4442656.3), 2^nd^ yr prairie (4442658.3), 20^th^ year prairie (4442659.3), 33^rd^ year prairie (4441281.3)). For all comparisons, we used a non-redundant protein database with an e-value cut-off of 10^−5^. We recognize that the ‘discovery rate’ for proteins may depend upon the efficacy of DNA extraction and the length of sequences that result, features that may vary among studies. Although we normalized the number of proteins identified with different metabolic functions by the number of proteins that were found per 100 fragments, we had no means of controlling for the different contiguous sequence lengths that occurred among different studies.

The mussel associated sequences are publicly available in the MG-RAST system under the following project identifiers (IDs 4441185.3 (tidepool), 4441191.3 (emergent,bench). The data in this manuscript and the analyses and comparisons to other public data sets are available via MG-RAST. MIGS/MIMS [30] compliant metadata describing the locations, sampling, data extraction and data is available in GCDML [31] format from within the MG-RAST system as well.

## Results

### Phylogenetic Analyses

For the tidepool mussel sample, there were 157,599 total DNA fragments with a total sequence size of 30,593,565 and an average sequence length of 194 bp. The bench mussel sample had slightly fewer contiguous sequences (141,293) from a similar sequence size of 31,304,272 and an average sequence length of 222 bp.

The BLASTX analysis against a non-redundant protein database matched 22.5% of the sequences in the tidepool sample of which 74% were bacterial and almost 3% were eukaryotic; the remaining 23% were unidentified. 1% of protein sequences matched to nitrogen metabolism. For the bench mussels, approximately 79% were bacterial with 2% eukaryotic and .4% Archaeal for the 25.6% that could be matched. Our protein ‘discovery rate’ of 22.5 and 25.6% was comparable or greater than other metagenome studies using 454-based sequencing technology [19], [29], but less than studies where direct library construction and sequencing was done [13].

**Figure 1:**
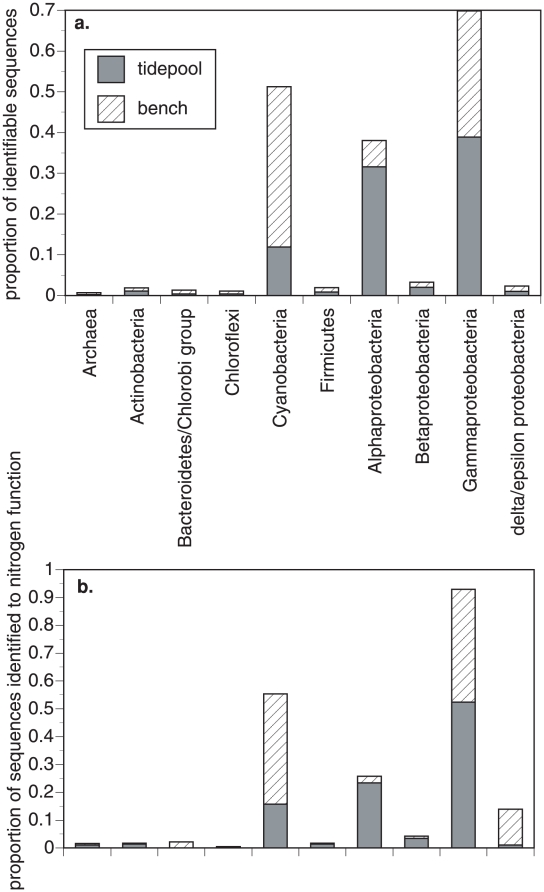
Taxonomic composition of surface-associated microbes of tidepool and emergent (bench) mussels. a. The relative representation of microbial phylogenetic groups in both the tidepool and emergent (bench) mussel samples based on shotgun pyrosequencing. Proportional representation is based on 157,599 total contiguous sequences for the tidepool mussels and 141,293 for the bench mussels. In b., the taxonomic composition as related to enzymes for nitrogen metabolism in Fig. 3.

**Figure 2:**
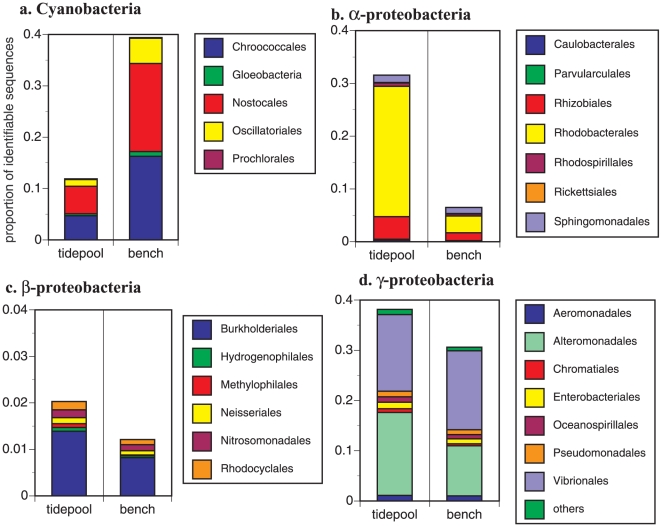
The relative proportional representation within the most commonly discovered bacterial orders on the surface of the mussel shells. a. Cyanobacteria, b. *α*-Proteobacteria, c. *β*-Proteobacteria, and d. *γ*-Proteobacteria among the tidepool and emergent mussel shells samples. Y-axes differ due to differences in relative abundance (see Fig. 1a).

When we compared the taxonomic composition of the 2 mussel samples to each other, they were similar at higher taxonomic organization, but differed slightly in the composition of lower taxonomic groupings (Table 1. Fig. 1a). Cyanobacteria, *α*-Proteobacteria and γ-Proteobacteria dominated both samples. The Cyanobacteria were more abundant on emergent mussels and were identified primarily as members of the orders Chroococcales and Nostocales (Fig. 2a). *Crocosphaera* and *Synechococcus* were identified in both samples, though more on emergent mussels. Both genera are photoautotrophs that are not thought to fix atmospheric nitrogen. The *α*-Proteobacteria were dominated by Rhizobiales and Rhodobacterales and included the nitrifying *Nitrobacter* (Rhizobiales) (Fig. 2b). There was an increased incidence of Rhodobacterales on tidepool mussels, including matches with *Rhodobacter* and *Roseobacter*, an aerobic anoxygenic phototroph. The β-Proteobacteria were highly similar between tidepool and emergent mussels and the nitrifying *Nitrosomonas* and *Nitrospira* were represented in both (Fig. 2c). The γ-Proteobacteria was the taxonomic unit with the greatest membership and was primarily composed of Vibrionales and Alteromonadeles (Fig. 2d). The ammonium oxidizing bacterium *Nitrosococcus* was represented in both samples. Although relatively few Archaeal proteins were identified, they included representatives of both Crenarchaeota and Euryarchaeota, and *Nitrosopumilus*, an ammonia-oxidizing chrenarchaeon, was detected in both samples.

**Table 1:** The taxonomic diversity of microbes from the surface of mussels in tidepools and on emergent benches.

		tidepool	bench
Archaea	Crenarchaeota	0.0003	0.0004
	Euryarchaeota	0.0020	0.0040
Actinobacteridae	Actinomycetales	0.0046	0
Bacteroidetes	Bacteroidales	0.0002	0
	Flavobacteria	0.0531	0.0757
	Sphingobacteria	0.0019	0.0005
Cyanobacteria	total	0.1020	0.0930
	Chroococcales	0.0464	0.0611
	Nostocales	0.0429	0.0232
	Oscillatoriales	0.0097	0.0079
	Prochlorales	0.0009	0.0009
Firmicutes	Bacilli	0.0017	0
	Clostridiales	0.0014	0.0002
	Mollicutes	0.0001	0
Alpha-Proteobacteria	total	0.3477	0.0450
	Caulobacterales	0.0021	0.0003
	Parvularculales	0.0010	0.0002
	Rhizobiales	0.0364	0.0019
	Rhodobacterales	0.2904	0.0335
	Rhodospirillales	0.0042	0.0005
	Sphingomonadales	0.0130	0
Beta-Proteobacteria	total	0.0125	0.0002
	Burkholderiales	0.0085	0
	Neisseriales	0.0009	0.0002
Gamma-Proteobacteria	total	0.3891	0.7724
	Aeromonada	0.0108	0.0045
	Alteromonadales	0.1655	0.1141
	Chromatiales	0.0076	0.0002
	Enterobacteriales	0.0126	0.0021
	Methylococcales	0.0015	0.0002
	Oceanospirillales	0.0106	0.0077
	Pasteurellales	0.0032	0.0002
	Pseudomonadales	0.0115	0.0002
	Vibrionales	0.1526	0.6429
Delta-Proteobacteria	Desulfovibrionales	0.0044	0
Fungi	Ascomycota	0.0040	0.0026

Proportions of the total identifiable sequences using the SEED protein database are given.

When we compared the emergent and tidepool mussels at the finest level for taxonomic affinities, only 7 identities differed between the 2 samples and all were single occurrences within 7 distinct phylogenetic groups (Crenarcheaota, Euryarchaeota, Actinobacteria, Chlorobi, Firmicutes, γ-Proteobacteria). Thus, the two mussel microbe assemblages were highly concordant in their overall composition, despite the fact that they came from different microhabitats.

When we compared the taxonomic composition of mussel shell microbes with other marine metagenomes, the dominance of γ-Proteobacteria in mussels and stromatolites, e.g. the nearshore sites, is apparent (Table 2). α-Proteobacteria were better represented in open ocean waters, though bench mussels had a large representation too. The representation by Cyanobacteria varied among sites with the 2 Line Islands of Fanning and Palmyra having high representation, though primarily by Chroococcales at Fanning (and on the bench mussels) and by Prochlorales at Palmyra. In contrast, the more oceanic Prochlorales were low in incidence on the mussel shells, represented by only 77 *Prochlorococcus* hits in each of the bench and tidepool samples.

**Table 2:** The comparative representation of bacterial and archaeal phylogenetic groups on mussel shells versus other marine systems with metagenomes analyzed by shotgun pyrosequencing and using MG-RAST and the SEED subsystem database (e<10^−5^)).

	tidepool	bench	Kingman	Palmyra	Fanning	Kiritimati	Georgia	Georgia	GS002	GS013	GS025	GS031
Crenarchaeota	0.000	0.000	0.000	0.000	0.000	0.000	0.000	0.001	0.002	0.002	0.003	0.010
Euryarchaeota	0.002	0.004	0.003	0.002	0.003	0.003	0.002	0.001	0.007	0.008	0.018	0.014
Actinobacteria	0.011	0.007	0.036	0.006	0.008	0.014	0.033	0.029	0.014	0.072	0.012	0.036
Aquificae	0.000	0.000	0.000	0.000	0.000	0.001	0.002	0.000	0.001	0.001	0.000	0.001
Bacteroidetes	0.063	0.130	0.134	0.076	0.065	0.047	0.030	0.029	0.083	0.091	0.036	0.102
Bacteroidetes/Chlorobi group	0.063	0.130	0.135	0.077	0.066	0.048	0.030	0.029	0.085	0.093	0.038	0.104
Chlamydiae/Verrucomicrobia	0.002	0.001	0.002	0.003	0.007	0.005	0.000	0.001	0.003	0.005	0.003	0.002
Chloroflexi	0.004	0.007	0.001	0.001	0.000	0.002	0.000	0.001	0.006	0.009	0.008	0.007
Cyanobacteria		0.394	0.088	0.331	0.356	0.117	0.015	0.020	0.023	0.022	0.253	0.019
Firmicutes	0.009	0.011	0.023	0.017	0.040	0.019	0.011	0.015	0.025	0.030	0.020	0.036
Fusobacteria	0.000	0.000	0.000	0.000	0.000	0.000	0.000	0.000	0.001	0.001	0.001	0.001
Planctomycetes	0.004	0.005	0.043	0.007	0.004	0.086	0.008	0.006	0.008	0.024	0.011	0.007
Alphaproteobacteria	0.316	0.065	0.378	0.211	0.059	0.093	0.245	0.255	0.505	0.455	0.082	0.334
Betaproteobacteria	0.020	0.012	0.024	0.011	0.006	0.023	0.106	0.137	0.028	0.043	0.023	0.053
Gammaproteobacteria	0.389	0.309	0.116	0.199	0.058	0.160	0.502	0.457	0.148	0.159	0.119	0.206
delta/epsilon proteobacteria	0.010	0.013	0.010	0.005	0.004	0.011	0.014	0.015	0.028	0.025	0.033	0.031
Fungi/Metazoa group	0.019	0.023	0.069	0.017	0.011	0.202	0.012	0.007	0.016	0.028	0.111	0.010
Viruses	0.003	0.002	0.008	0.003	0.009	0.010	0.002	0.017	0.105	0.044	0.208	0.020

Four islands in the Line Islands system (19; ref numbers: 4440039.3, 4440041.3, 444027.3, 4440037.3), 20 L of Georgia coastal seawater that was incubated with 2 components of dissolved organic carbon (29;DMSP, 4440360.3, VAN, 4440365.3), and 4 samples based on 200 L surface water from the GSOE program (13; GS002 = Gulf of Maine (4441579.3), GS013 = Nag's Head, NC (4441585.3), GS025 = Cocos Island (4441593.3), Costa Rica, GS031 = upwelling zone off of Fernandina, Galapagos (4441597.3)).

### Metabolic Analyses

Our mussel associated metagenome analysis found many matches to proteins in the non-redundant database relevant to metabolic functions (Table 3). The relevant ranking of metabolic functions was strikingly similar to Dinsdale et al.'s [18] ranking based on the mean of 45 microbial metagenomes from habitats as diverse as the digestive systems of animals to a coral holobiont.

**Table 3:** Percentage of sequences that matched major metabolic categories (Subsystem Categories [17]) using the SEED non-redundant database for both tidepool mussels and emergent, bench mussels and compared with a mean value for other microbial metagenomes from a variety of species and systems (from [18]).

Metabolic category	tidepool mussels	bench mussels	Other microbial metagenomes
Carbohydrates	11.35	12.67	17.218
Amino Acids	8.15	8.91	12.036
Virulence	7.50	6.44	9.788
Protein metabolism	6.15	7.32	9.123
Respiration	4.07	4.48	7.139
Photosynthesis	1.26	0.94	6.965
Cofactors, Vitamins, Prosthetic Groups, Pigments	7.28	6.42	5.411
RNA Metabolism	3.90	4.25	3.971
DNA Metabolism	4.03	3.63	3.970
Nucleosides and Nucleotides	2.50	2.94	3.316
Cell Wall and Capsule	3.90	3.77	3.235
Fatty Acids and Lipids	1.60	1.27	3.095
Membrane Transport	2.55	2.51	2.736
Stress Response	2.64	2.45	2.599
Cell Division and Cell Cycle	2.06	1.41	1.791
Nitrogen Metabolism	1.06	1.04	1.547
Sulfur Metabolism	0.95	1.02	1.230
Motility and Chemotaxis	2.20	2.34	1.022
Phosphorus Metabolism	1.69	1.55	0.909
Cell signaling			0.885
Potassium metabolism	1.21	1.21	0.796
Secondary Metabolism	0.09	0.04	0.159

We hypothesized that enzymes related to ammonium assimilation would be present in mussel shell microbes as a means of utilizing the ammonium excreted by mussels. When we used the protein database to match to metabolic function, we found 1.0% of the sequences in each sample matched to nitrogen metabolism, with a total of 446 sequences found in the tidepool mussels and 445 in the bench mussels. The distribution of sequences associated with different aspects of nitrogen cycling were relatively similar among the 2 samples (Table 4), and included not only ammonium assimilation, but also nitrate and nitrite ammonification, allantoin degradation and nitric oxide synthase as the dominant metabolic components. Enzymes such as ammonium monooxygenase subunit A (*amo*A) and glutamine synthetase were also detected. Denitrifying enzymes were also present, while nitrogen fixation (as indicated by nitrogenase) was nearly absent.

**Table 4:** Number of sequences associated with nitrogen metabolism using the SEED database.

	tidepool mussels	bench mussels
Nitrogen fixation	3	1
Nitrosative stress	16	12
Nitrate and nitrite ammonification	178	133
Ammonia assimilation	98	130
Cyanate hydrolysis	14	22
Dissimilatory nitrite reductase	12	6
Nitric oxide synthase	16	60
Allantoin degradation	39	41
Denitrification	51	27
Nitrogen fixation	3	1
Nitrosative stress	16	12
Total	446	445

Figure 2b shows the taxonomic affiliation of nitrogen cycle enzymes.

The taxonomic affiliations of the enzymes involved in nitrogen metabolism bore strong similarity to the overall representation of Bacteria and Archaea in the samples (Fig. 1b). Thus, all major groups of microbes contributed to nitrogen metabolism in approximate proportion to their abundance, although Delta-Proteobacteria were better represented in the bench mussels and Cyanobacteria and Gamma-proteobacteria were also strongly associated with nitrogen metabolism.

**Figure 3:**
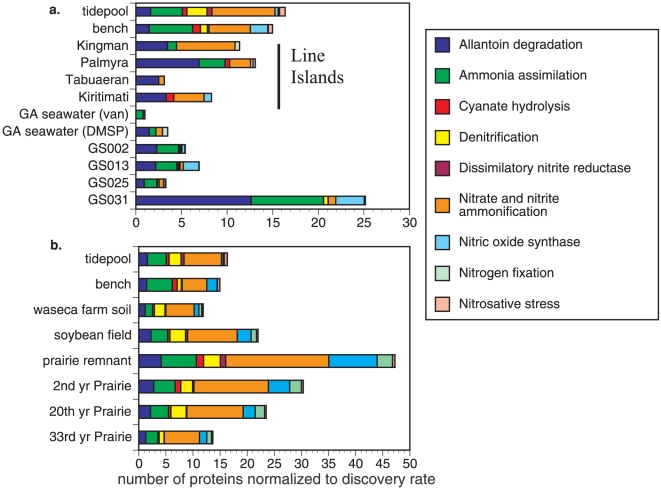
The number of proteins matched to nitrogen metabolism functions among multiple metagenome studies. a. marine metagenomes and b. soil metagenomes. To facilitate comparison among studies, the number of matches is normalized to the ‘discovery rate’ for proteins in the dataset (number of protein matches per 100 fragments). For a. the marine metagenomes are as in Table 2, while b. uses Midwestern soil metagenomes (MG-RAST ids 4441091.3, 4442657.3, 4442656.3, 4442658.3, 4442659.3, 4441281.3, respectively).

Nitrogen metabolism enzymes in the mussel shell microbes show a strong pattern for much uptake and transformation of inorganic nitrogen especially ammonium uptake and ammonification, a pattern shared with some of the Line Islands metagenomes and also with the waters surrounding the Galapagos upwelling region (Fig. 3a). Other regions were comparatively depauperate in proteins for nitrogen function, including seawater from areas adjacent to Georgia, Maine, North Carolina and Costa Rica. Nitrogen fixation was suggested to be relatively minor in these areas, excepting the Georgia VAN sample. When using MG-RAST to test the hypothesis that our mussel associated microbes would show strong similarity with soil metagenomes from current or former agricultural fields of Illinois, the similarity of enzyme types was marked (Fig. 3b). In these soils, as in association with mussels, enzymes related to ammonium uptake or nitrite and nitrate use were particularly well represented, though soils had an increased incidence of enzymes related to nitrogen fixation.

**Figure 4:**
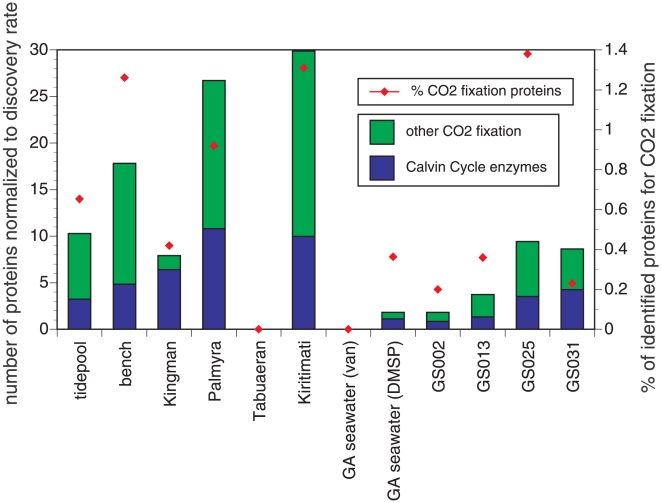
The number of proteins involved in CO_2_ fixation, including those of the Calvin-Benson cycle among a selection of marine metagenomes. To facilitate comparison among studies, the number of matches is normalized to the ‘discovery rate’ for proteins in the dataset (number of protein matches per 100 fragments). The point symbols represent the percent of all proteins identified that are used in CO_2_ fixation.

Proteins related to CO_2_ fixation were also well represented in the mussel shell samples, including enzymes of the Calvin cycle (primarily RUBISCO) and exceeded those in Georgia waters and the 4 Global Ocean samples (Figure 4). We matched 232 CO_2_ fixation-related proteins in the tidepool sample (0.65% of all proteins) and nearly twice that in the bench sample (456 for 1.26% of all proteins). The Line Islands had a variable amount of CO_2_ fixation proteins with Palmyra and Kiritimati (Christmas Island) having the greatest number. The percent of proteins identified for CO_2_ fixation out of the entire protein pool of all metagenomes ranged between 0.0 and 1.38 across all metagenomes.

## Discussion

The microbes on mussel shells showed both a taxonomic and functional composition that reflects a nitrogen-rich environment. The major nitrifying bacterial genera that are described (*Nitrosococcus*, *Nitrosomonas*, *Nitrospira*, *Nitrobacter*, [32]) were found in both samples, as well as some Crenarchaeota (*Nitrosopumilis*, [33]). Ammonium assimilation enzymes were also well represented in both samples. Denitrifying genera were also identified, including *Shewanella* and *Roseobacter*, as well as enzymes related to denitrification including nitrite- and nitrate-reductases and all were more prevalent in the tidepool rather than bench mussels. Although the presence of denitrifying enzymes and genera suggest that this process may be occurring in low oxygen microsites in tidepools, its signature feature, the uptake of nitrite is not suggested by water nutrient sampling within tidepools [6]. Whether anaerobic ammonium oxidation (anammox) is important here is unclear. We found no matches to the putative species or enzymes thought to be important to anammox [34], but recognize that our detection may be limited by the relatively little that is known about anammox metabolism. However, as with denitrification, these environments are typically well-oxygenated and we might not expect anammox to be highly important. Although Cyanobacteria and many genera from Proteobacteria that are known nitrogen fixers were represented in our mussel shell microbe samples [35], nitrogenase enzymes were absent, suggesting that these are photoautotrophs that do not fix atmospheric nitrogen. The rich variety of other nitrogen sources in this nearshore environment may select against nitrogen fixation or result in competitive inferiority of nitrogen fixers compared with ammonium or nitrate utilizing microbes, a pattern described in plankton assemblages [36] [Bibr pone.0010518-Montoya1]–[38]. The potential for relatively high ambient availability of ammonium can be illustrated using Suchanek's [25] estimate of a mean number of 4661 mussels per m^2^ on Tatoosh Island coupled with per mussel excretion rates [39]. Using both, we estimate >3 g of ammonium (∼55 mmol) excreted per day per m^2^ of mussels, a substantial input of inorganic nitrogen. If rates were known for other invertebrates and vertebrates in this system, such as seabirds and marine mammals, this input would likely be much higher. Nitrate from upwelling is also typically high, and is at concentrations of approximately 20 µM at Tatoosh and nearby sites during spring and summer months [6], [9]. In sum, the nearshore in proximity to *M. californianus* mussel beds are rich in inorganic nitrogen and may provide an environment that selects against the persistence of nitrogen fixing organisms.

Our comparison of nitrogen metabolism among different systems further substantiated the diverse nitrogen functions on mussel shells. The Line Islands in the South Pacific, the coastal ocean samples of Georgia and the Global Ocean Sampling Expedition, excepting the upwelling region off the Galapagos (Fernandina, GS0031), all had fewer enzyme matches for ammonium assimilation. In terms of the distribution of enzymes related to nitrogen metabolism, mussel shell microbes had much in common with the upwelling region of the Galapagos and Midwestern soils, including a range of different metabolisms with ammonium assimilation and ammonification predominating, though nitrogen fixation was much more common in these soils than indicated for the surface of mussels. The mussels, Fernandina, and the Midwestern soils likely have a rich nitrogen environment that promotes similar microbial opportunities. We note, however, that although all these metagenome studies were analyzed with a common platform (MG-RAST and SEED), comparisons among metagenome studies to date should consider differences among studies in extracting and sequencing. All but the Global Ocean Sampling Expedition used similar shotgun sequencing methodologies. However, the length of reads in the Line Island and Georgia samples were ∼100 bp (Roche GS-20) compared with the ∼200 bp and greater length in this mussel study and in the soil metagenomes (Roche GS-FLX). The cloning and Sanger-based sequencing approach used by the Global Ocean Sampling program (GS002,GS013,GS025, GS031), however, generated longer sequences (∼1000 bp) and thus may have a higher protein discovery rate, although cloning bias would also be a factor.

The microbial assemblages of pool and bench mussels were very similar taxonomically and functionally, indicating that previous results suggesting nitrification in tidepools is probably a phenomenon general to association with mussels regardless of habitat. These few differences have some interesting implications. For example, Cyanobacteria were more abundant on emergent mussels. In the absence of evidence for nitrogenase, it is possible that these are endolithic phototrophs of mussel shells. Research with other mussels (*Perna perna*) have shown a detrimental effect of Cyanobacteria that are phototrophic endoliths [40], [41]. Although these endolithic forms appear poorly described taxonomically and ecologically, the possible increased incidence on emergent rock suggests that different environmental conditions may affect the distribution of these microbes. The reduced relative composition of Cyanobacteria in tidepools was possibly compensated for with α-Proteobacteria in tidepools, particularly Rhodobacterales, a group referred to as primary surface colonizers [42]. The high density of molluscan grazers in tidepools and their near continuous opportunities to graze might suggest some grazer tolerance or resistance on the part of these α-Proteobacteria, a result supported by experimental grazer removals in marine benthic systems [43]. In terms of nitrogen metabolism the tidepool and emergent bench mussels were very similar with tidepools having only a slightly greater incidence of ammonification.

Some compositional differences between mussel shell microbes and other marine metagenomes was marked. For example, our mussel shell microbes and the Georgia coastal waters were dominated by γ-proteobacteria; dominance by γ-proteobacteria has also been demonstrated in association with the Caribbean coral *Porites astreoides* [44]. In contrast, open ocean samples such as those from the Sargasso Sea [17] and the locales of the Global Ocean Sampling Expedition (Fig. 3, [13]) were dominated by α-Proteobacteria, a group associated with photoautotrophs of the open ocean. The Cyanobacteria were also variable in abundance, with the bench mussels and the Costa Rican sample (GS0025, Cocos Island) having a relatively large proportion of Cyanobacteria. However, the composition of the Cyanobacteria differed among these sites. The Costa Rican waters were dominated by the order Prochlorales (genus *Prochlorococcus*), a group that had only 77 contigs per sample in the mussels, a meagre ∼.14% of all identifiable sequences. Although *Prochlorococcus* is known to be an abundant cyanobacterium in the open ocean where it can comprise as much as half of the photosynthetic biomass [45], it did not dominate in this nearshore environment.

Microbial activity related to the carbon cycle also appears to be a strong feature of the assemblage on mussel shells. There were as much or more proteins identified with CO_2_ fixation for the mussel-associated metagenomes as there were for any of the other marine metagenomes studied, suggesting that microbial nitrogen and carbon cycling are prevalent on these shells. Although there are at least 5 possible microbial pathways for CO_2_ fixation by microbes [46], the Calvin cycle is likely to be the most prevalent, based on the abundance of Proteobacteria and Cyanobacteria in these samples. The relative low incidence of microbes associated with other carbon fixation pathways (green sulfur bacteria, Chloroflexi) and the aerobic nature of the environment, make anaerobic and anammox pathways less likely.

All the described taxonomic and metabolic diversity came from a surface sample of only 6 mussels in an area where mussels can number in the thousands per square meter, and thus indicates the quantitatively significant role that mussels may play in microbial transformations for the nearshore nitrogen and carbon cycles. We acknowledge, however, that we have no water column censuses nor analyses of other substrates and cannot exclude the possibility that other substrates also serve as nitrogen transforming areas. Further genetic analyses in this system are thus warranted. Whether mussels are alone or not in providing suitable habitat for these microbial populations, the genetic data presented in this study suggests that if nitrogen is continually recycled and transformed by this microbial assemblage, then this provides a significant mechanism for the retention of nitrogen in nearshore areas, thus ameliorating the advection of nitrogen during upwelling events. Whether mussels are a unique node for microbial and biogeochemical activity, or one of several, the threats to their persistence are numerous and include declining ocean pH [47], low oxygen events [48], changing thermal environments [49], anthropogenic nitrogen pollution [50], and toxic algal blooms [51]. Although the work summarized here adds to our understanding of the interaction between macrofauna and their microbial associates, it also underscores how little of this diversity has been described in habitats that are otherwise well characterized in their ecological dynamics.
